# Pathological Physiology

**Published:** 1868-01-01

**Authors:** 


### CHICAGO MEDICAL JOURNAL.

*Vol. XXV.—JANUARY* 1, 1868.—*No.* 1.

### PATHOLOGICAL PHYSIOLOGY.

### THE CONTINUOUS VENOUS MURMURS IN THE NECK.

*Clinical Lectures delivered at the Hospital "La Charitè," by M. Monneret, Professor of Clinical Medicine to the Faculty of Medicine, Paris.*

TRANSLATED EXPRESSLY FOR THE JOURNAL, BY WALTER HAY, M.D., CHICAGO.

GENTLEMEN:—This clinical lecture, which will be the last, suggests the approach of vacation, and the paucity of subjects. I shall devote myself to treating, for your benefit, a question at once theoretical and practical, reviewing as clearly as possible a doctrine which is based upon numerous experiments, and upon facts which it has been easy for you to establish daily at the bedside of the sick.

It concerns the continuous murmurs which have their seat in the neck, and particularly on the right side. I will point out to you hereafter their cause.

The extreme frequency of these sounds, and the progress which the theory of their mode of production has made, render their clinical study necessary.

I do not intend to give you a very complete history of them, but in consequence of errors imprinted in certain books, and in recent memoirs, it is right that I devote myself to making you acquainted with the truth, from the opinions of men who have best studied these phenomena, and have given a clear theory of them, founded upon the laws of acoustics, upon ideas received, especially respected, and in relation with numerous experiments, with undeniable facts, whose interpretation can not be permitted to be changed.

Two sorts of sounds occur in the vessels of the neck: an intermittent and a continuous sound; the continuous sound, with swells, appears to be nothing else than the continuous sound duplicated with an intermittent one.

Now, under the reign of Læennec—and his opinions prevailed a long time after him—it was conceded that all these sounds could only be produced in the arteries, in the heart, and in the large vessels proceeding therefrom.

Ogier Ward, in 1837, was the first who expressed the opinion that the vascular sounds of the neck were continuous sounds, which occurred in the veins, and not in the arteries. Almost as soon, Hope adopted his theory, and gave to it the success which could be impressed upon it by a man who had so great a renown in the history of diseases of the heart. This theory became universal in England.

Aran was the first in France who rallied to the support of this theory in 1843. After him, I may consider myself as the one who has studied it most completely in all its details, and taught it, in the year 1847. At this epoch it occurred to me, for reasons drawn from the laws of acoustics, and from the works of Savart and Cagnard-Latour, that there could be a continuous sound only in the veins.

Hence a series of experiments, which I instituted since the year 1847, at the Hospital Bon-Secour, with the view to determine the influence which the sanguineous fluid, put in motion in a tube, could have upon continuous sonorous vibrations, and especially the influence of the composition of the liquid upon the intensity of these vibrations.

The results at which I arrived, furnished matter for four memoirs, which appeared consecutively in the "Medico-Chirurgical Review" of 1850, in March, April, July, and August. All this work was especially designed to prove that the continuous vascular murmurs of the neck — the only ones about which there can be any question — have their seat in the large veins of the neck, and their cause in the liquid which flows through them; that is to say, in the blood, of which the composition may vary.

A sound is produced each time that the sanguineous fluid moves in the vessel with a certain speed, moistening its walls, as in anæmia.

This sound is by so much the stronger as the blood is the poorer in globules. Aside from anæmia, and from diminution of the globules, there is never any murmur. As we shall see that the diminution in the globules produces necessarily an augmentation in the serosity, even as far as 100 to 200 parts in 1,000 parts of blood. We can understand that all the sanguineous liquid so altered by the serum should produce a continuous sound in the veins, where alone the blood flows with sufficient rapidity and continuity, near the outlet of the vena cava, to produce this sound.

It is a grave error to say that plethora can engender it. It is necessary to know how to distinguish true from false plethora. Since the date of the memoir above mentioned, I have never revoked my opinion; I have constantly maintained it in my course of internal pathology in the school of practice where I have passed in review every theory. Later, in 1861, I reproduced, with renewed assurance, with new force of conviction, this same idea that the continuous sound could only have its seat in the veins, in the heart, at the mouths of the great veins of the neck, and that it could only develop itself there, under the influence of an anatomical arrangement upon which I shall insist hereafter. I desire to establish firmly the point, in order that you may perceive that during nineteen years I have always expressed the same opinion, based upon the experiments of physicians who have promulgated the true laws of acoustics.

Savart and Cagnard-Latour did not hesitate about the mode of production of these venous murmurs; they understood, at once, the necessity of applying to the region of the neck, the propositions which they had formulated for liquids foreign to the economy. Moreover, we can find no explanation more lucid than that which they have given.

After what has just been said, you will, perhaps, be astonished, upon opening a memoir recently published by M. Parrot, in the "Archives of Medicine" for July, 1867, to find these assertions counter to those which I myself have sought, not because I am their source. It is not I who am the inventor of the theory which I sustain, but because it is just to refer to their true authors, the ideas which they have promulgated. I would remark to M. Parrot that he is completely in error, when he asserts that Chauveau, and then Marey, are the only authors who have sought to attribute the venous murmurs to the composition of the liquid, and the diminution of tension of the vessels.

This is false. The memoirs already mentioned of 1850, reproduce minutely all the experiments which have been made, in order to account for the bellows sounds of the veins. They point out their causes much before these two authors, I will say more, at an epoch long anterior to them.

Savart, in 1830, Cagnard-Latour in 1833, had published the laws which govern the production of these murmurs, by building upon acoustics, theories the most clear and most sure.

These theories are accepted by all, not only in medicine, but in physics; no one is ignorant of them, I will not, therefore, dwell upon them.

The Experiments, published by Dr. Laharpe, in "L'Archives Generales de Medicine," have for their subject the influence of the composition of different liquids upon the production of sounds, and we find there, studied with extreme detail, every thing concerning the vibration of liquids according to their composition, upon which he makes the sounds to depend.

I come now to the study of these sounds, but before going further I must point out to you two conditions which are essential to them: The one is their normal anatomical state, the other their pathological.

Recall what you already know, that the continued vascular murmur exists only on the right (it is clear that I do not speak of arterio-venous aneurisms), to the serious study of which, moreover, I have devoted myself in a memoir presented to the Surgical Society upon the subject of an arterio-venous perforation formed in the popliteal space. There were two sounds, one a continuous sound, the other a swell, and I demonstrated that the continuous sound occurred in the vein, whilst the swell sound was nothing else than an intermittent bellows murmur which occurred in the artery.

I repeat, then, that in the region of the neck, the venous murmur occurs always on the right; if by chance it is not so, and the sound makes itself heard on the left, it is by transmission, and because it is then sufficiently intense to transmit itself from that side. The stethescope detects very readily upon the left side the sound which is formed on the right. I have, moreover, never found this sound elsewhere, neither over the arteries nor the veins of the lower extremities, nor over the other large vessels.

In the next place the vascular sound is the effect of an alteration occuring in the composition of the blood.

Every time that, in a healthy individual, and who consequently exhibits no vascular sound, the blood becomes altered in such a way that it moistens the internal walls of the veins, it produces a sound. The liquid begins to speak. To sum up: The alteration of the blood engenders a sound, a special anatomical arrangement gives origin to this sound, and to localize it in the right side.

It is this anatomical arrangement which I shall now attempt to discuss in a few words.

Three aponeurotic planes exist in the neck. The first, the most superficial, is easily seen in subjects who have little fat. The external jugular rests upon this aponeurosis, in the upper three-fourths of its extent; below this it penetrates it, in order to return into the subclavian fossa, on the internal surface it unites in order to empty itself into the subclavian vein. It results from this, that this vessel, sustained by the aponeurosis, is continually distended in certain positions which are given to the head in turning it; then the sanguineous fluid rushes through it with its maximum speed. Thus, this membrane should be stretched in order to produce the vibratory trembling, a phenomenon completely parallel to the venous sounds, and derived from the undulatory vibrations.

The second plane is known under the name of middle or omo-hyoid aponeurosis; it connects the two scapulo-hyoid muscles, and is inserted into the sternum, and into the inner border of the clavicle, enclosing in its reduplication the muscles of the sub-hyoid region, and the anterior jugulars.

According to Mr. Richet, who has described with particular care the middle aponeurosis of the neck, this membrane sends out from its lower surface, fibrous prolongations, which attach themselves to the trunks of the right and left brachiocephalic veins, and fix them to the upper bony rim of the chest. It receives then, in a reduplication, at the level of their opening into the subclavian vein, the internal and external jugular veins.

M. Richet, by reason of this arrangement, assigns to the extensor muscles of the omo-hyoid aponeurosis, the function of maintaining distended all the great veins of the region, and of thus rendering the circulation as easy as possible.

The third plane is that which exhibits the most intimate and most important connections with the vessels of the neck. It assumes an arrangement entirely peculiar, whose connections have been very recently clearly displayed by M. M. Ledentu and Lannelogue, who have had the merit of demonstrating clearly the existence of these aponeuroses, and their arrangement, which it is easy to comprehend by casting the eye over the anatomical specimens prepared by the authors whom I have just named, with this intention.

An aponeurotic layer detaches itself from the lower edge of the thyroid-body, descends in front of the trachea, passes behind the sternum, in front of the left brachio-cephalic vein, and is inserted upon the anterior face of the pericardium.

Laterally this fibrous leaf is inserted into the internal concave border of the first rib, and higher up it throws itself upon the internal jugular veins of which it forms the enveloping sheath.

But whilst the sheath is simply cellular above the middle part of the neck, in the lower part of that region it is very resistant at the confluence of the subclavian and the right internal jugular, where it forms a fibrous ring adherent to the first rib by one part, to the veins by the other. Not far from this confluence the external jugular empties into the subclavian. This one adheres equally to the former fibrous plane.

This costo-pericardiac ligament is then the true fibrous band which keeps distended the openings of the veins of the lower part of the neck. Moreover, from the lateral part of its deep surface, near its insertion into the first rib, originate two fibrous ligaments, which attach themselves to the brachio-cephalic venous trunks, even in the thorax, and playing, relatively to these veins, the part of suspensory ligaments, preventing their walls from collapsing during the thoracic inspiration.

The inferior thyroidal veins, also perforate this ligament, which forms for them a sort of fibrous semi-canal at the level of their outlet.

To sum up: there exists in the neck, and it is this point which I wish to bring prominently before you, a resisting plane, which sustains the whole venous mass, and maintains distended the superior vena-cava, the right and left brachio-cephalic venous trunks, and the confluence of these two trunks, which is situated to the right and not to the left, exactly in the direction of the brachio-cephalic trunk of that side, which is continuous directly, it should be remembered, with the vena-cava superior.

It is towards this confluence, cylindrical, regular, always distended, that the blood precipitates itself with rapidity, even as far as into the auricle, from the superior parts of the head and neck. It is here that all the sanguineous molecules, precipitated from the superior part, oscillate, vibrate, and at the same time produce this venous murmur which you hear principally to the right and behind the clavicle, at the head of this sort of general outlet, in which they vibrate and speak the liquid molecules agitated in so many different senses. These vibrations transmit themselves to the upper parts; and thus it is that we can, under the form of sound and of hydraulic vibrations, perceive them at the left side, and especially as far as the middle and upper right side of the neck. It is doubtless unnecessary to recall to you that the transmission of vibrations is effected to an enormous distance, especially in the direction of a liquid current, and that the blowing murmurs of a large pectoral artery is still very perceptible in the popliteal region, and even to the foot. In the experiments which have been made upon this subject, there has been much room for astonishment at this power of transmission, and it thus often becomes a cause of serious difficulty in the detection of the location of murmurs. Such is the entire physiological explanation of the production of sounds; an aponeurotic arrangement accounts for it perfectly, and it is very easy to comprehend why the vibration does not produce itself at the left, and why it is heard there only by transmission.

Let us return now to the second condition which we have to examine, to the influence which the composition of the blood exercises upon venous sounds.

When this liquid, I have already stated to you, is modified in such a manner that it wets the walls of the vessel which encloses it, its flow is accelerated, and a sound is produced. It has been demonstrated perfectly that the intensity of the murmur produced by venous liquid is proportional to the rapidity of the flow of the liquid, and the more readily the liquid moistens the walls, and the more rapidly it flows, the more intense is the murmur; likewise the more aqueous the blood, the less dense is it, and the stronger is the venous blowing. It will appear, especially in all its intensity, if you augment the rapidity of its flow by stretching the aponeuroses of the neck by a favorable position. Then, also, there can be felt with the finger the peculiar thrill to which I shall revert, and which consists in a series of undulations which transmit themselves to the ear, by a continuous sound with swells, or by musical sounds to which different appellations have been given.

If it is desired to analyze these murmurs, it is perceived that they consist of two sounds, the one continuous, the other intermittent, of a uniform tone, which is dominant, and of a modulated tone which varies incessantly. It is easy to give a clear and natural explanation of this phenomenon. When a cord is made to vibrate upon a sounding-board, and sand is caused to fall upon this latter, then are produced swellings and constrictions, with the same note; the same thing takes place in a liquid which vibrates. Two sorts of vibrations are observed—the one uniform, the other intermittent, abrupt—hence the modulated murmur of the arteries, the murmur de diable (devil), de souffle (bellows), and other names more or less incorrect, which are made use of to designate these sounds. In order to be familiarized with these phenomena, and to have the satisfaction of hearing sounds the most varied and agreeable, it is only necessary to place the ear upon a tube in which a liquid is caused to flow, whilst varying the velocity of its flow, there are reproduced, at once, the most varied intonations. They are recognized in the sounds so varied of this hydraulic organ, whose tones often surpass in beauty those of the wind-organ. I will not dwell upon all these facts, which belong rather to the domain of acoustics than of medicine.

The vibratory thrill, which accompanies the venous murmur, is perceptible to the finger. It imparts a sensation similar to that which is experienced when a cord, making solid vibrations, is touched; the liquid which flows and vibrates in the vein may be accurately compared to this vibrating cord. The vibratile thrill is nothing else than the series of solid undulations of water or of blood transmitted directly to the hand. The continuous, modulated, venous sound, or that with continuous and intermittent strokes, is the sound which this same solid undulation produces and determines.

There is, then, always certainly to be found united at one and the same point the sound and the vibratile thrill.

The phenomena which I have just indicated, and the interpretation which I have given of them, are so real that they can be reproduced upon the dead body.

Censure has recently been cast upon the comparison sought to be established between the venous sound produced experimentally upon a dead body, and that which is observed in the living. I am sorry for those who find in this neither resemblance nor pleasure; for it is identical both in sound and tone. To deny this identity is to prove that the endeavor to reproduce these experiments has not been made.

When we are so fortunate as to be able to reproduce upon a dead body, with perfect exactness, the phenomena which occur in the living, we are right, it appears to me, in deciding upon the identity of the cause which produces them.

Open the jugular or the carotid of a dead body, fit into the vessel a tube of caoutchouc, which communicates with a reservoir above furnished with a stop-cock: in order to determine a continuous musical sound, it will suffice to open, more or less freely, the vessel, and to give passage to the liquid, after it is certain that the flow is effected with facility. When, in order to increase or diminish the velocity of the flow of the liquid, the body is inclined, at the same instant there is heard a sound very intense and modulated, which is nothing else than the venous sound which has just been produced.

Not only is the sound heard, but also the molecular vibrations are perceived, of which Cagnard-Latour has indicated the mechanism, and which he has designated under the name of rotary molecular murmur.

These vascular murmurs may be imitated so well that they may be listened to for a long time and with pleasure. The true causes of the murmur are apparent to the eye. If it is desired to render the murmurs continuous or intermittent, it suffices to render the flow more or less rapid: if it is desired to render it more or less strong, it suffices also to increase or diminish the velocity of the current, and that is easy, it being necessary only to vary the inclination of the body or the pressure of the liquid.

If the body is horizontal, a feeble murmur is heard; if, on the contrary, the inclination is such that the speed may be considerable, the murmurs are so intense that they offend the ear, and the more rapidly the liquid flows the more the intensity of the murmur augments, the more decided becomes the tone, and the more intense the vibratory thrill.

In these experiments it is also perfectly easy to verify the other proposition which has been announced: the intensity of the sound is directly proportional to the velocity of the flow of the liquid, and inversely proportional to the opening made in their walls. That is to say, the more rapid the flow the louder the sound, and that, on the contrary, the more contracted the orifice the less intense the murmur.

These are indisputable facts, which admit not the least doubt. Physicians unconvinced by experiment should decide upon them. Certain authors interpose a third cause in the production of vascular sounds; they assume that the wall of the vessel is a little flaccid, loosened, relaxed, and that there are formed in its interior little projections, folds, wrinkles, which originate the continuous venous murmurs; without these little obstacles they can not comprehend the possibility of a murmur. They are completely deceived. In 1850 I was carried away very decidedly, without being committed to it, towards this theory of flaccidity. Now I have come around to this position, that, in science it is always permitted to modify opinions, and to lay aside that false pride which urges one never to say no, when he has once said yes.

In examining anew the theory of which I speak, or rather this modification of it, which appeared to me sufficient to explain these vascular murmurs, I discovered that it was impossible; and moreover no one has demonstrated this pretended flaccidity of the vessels of the neck, even among chloro-anæmic subjects it has been assumed gratuitously.

Savart, whose authority in physics can never be annulled, after experiments of equally extreme delicacy and precision, by reason especially of the acoustic capacities of his ear, which enabled him to notice intonations, modifications of sound, where no one else would ever have perceived any, had arrived at the belief that the flaccidity of the walls, which receive and transmit, renders them more fit to receive and transmit, but not to produce them. He has proved it by augmenting and diminishing the tension of a membrane; by producing a detached sound, he remarked that the intensity of the vibration increased when he relaxed the membrane, and that it became, on the contrary, more and more feeble as he stretched it, as is seen in the membrane of the tympanum when prepared to receive feeble sounds. There is then a tendency in flaccid membranes to vibrate more readily, in unison with sonorous bodies placed in their vicinity.

The venous walls could enter into a state of vibration if they were free and stretched; but it is sufficient to ruin this doctrine, to notice that the venous membrane is every where adherent by one of its surfaces to the surrounding tissues.

Moreover, it asserts that the walls of the veins in chlorotic and anæmic subjects are far from being flaccid, and especially recall this principle, that there is no vacuum in the organism.

Bleed an animal to whiteness, he falls inanimate; return the blood to the brain by suspending him by the hind legs, the animal revives immediately; if it is left in this position, reparative nature carries the fluids from every point in the organism into the vessels, effects the re-absorption of the serosity of the different fluids, and thus brings them back into the circulatory system. Open now the vessels, the circulatory system is found as full as before; the loss of blood which has been sustained is hardly perceptible. The same thing occurs in the case of one wounded, who has lost a large quantity of blood; as also in that of the parturient woman who has sustained a considerable metrorrhagia. Subjects so anæmic as to be considered dead, nevertheless returned to life, and soon after their veins were found distended with blood.

After a copious bleeding the vessels are still full and distended; the blood is, however, much paler, because the globules are diminished. There is no vacuum in the organism, and especially in the circulatory system, since another liquid comes immediately to replace the blood; flaccidity can not exist.

I have already stated that Savart had demonstrated the relations of consonance of a relaxed membrane, with one which is stretched vibrating in unison; and this consonance any one may establish for himself, when, in a theater, for example, one attempts to appreciate the most delicate tones. The membrane of the tympanum is then relaxed, and unconsciously the attempt is made to harmonize the most feeble vibrations which come to the ear. In a word, the tympanum has been rendered more flaccid, as Müller and many other physiologists have demonstrated.

But, as has been said already, this flaccidity can not be admitted for the veins of the neck. The walls of the veins are not free; one surface adheres to the aponeurosis, the other is in immediate contact with the blood.

Now Savart always made his experiments upon membranes with a free surface, and the walls of the veins of an anæmic subject should be flaccid, in order that they might be able to vibrate.

It may be objected, it is true, that the flaccidity does not exist, but that there is a liquid which transmits to them immediately sonorous vibrations which originate there. That would be possible indeed if they could originate exteriorly; but they originate in the vein from the collision of the sanguineous molecules.

(*To be continued.*)

